# Enzymatic and toxigenic ability of opportunistic fungi contaminating intensive care units and operation rooms at Assiut University Hospitals, Egypt

**DOI:** 10.1186/2193-1801-2-347

**Published:** 2013-07-29

**Authors:** Mohamed Bassam Aboul-Nasr, Abdel-Naser Ahmed Zohri, Enas Mahmoud Amer

**Affiliations:** Faculty of Science, Department of Botany, University of Sohag, Sohag, Egypt; Faculty of Science, Department of Botany, University of Assiut, Assiut, Egypt

**Keywords:** Airborne fungi, Enzymes, Mycotoxins and pathogenicity

## Abstract

Total of 110 isolates belonging to 8 fungal species collected from intensive care units (ICUs) and operation rooms (ORs) at Assiut University hospitals were examined for their ability to produce some extracellular enzymes and mycotoxins which are considered as important factors involved in for fungal pathogenicity. The results revealed that 73, 92 and 78 out of the 110 tested isolates produced protease, lipase and urease respectively; meanwhile, 77 of the tested isolates exhibited some hemolytic activities. Chromatographic analysis (TLC) of the crude extract of the fungal isolates tested revealed that 79 isolates of them had the ability to produce at least one of these mycotoxic compounds (aflatoxins B_1_, B_2_, G_1_, gliotoxin, fumigillin, T-2, zearalenone, roridin A & E, verrucarin A & J, trichoveroids, satratoxin H & E). These results demonstrate that the opportunistic fungal species isolated from (ICUs) and (ORs) and tested exhibited some enzymatic and mycotoxic ability which are the most effective virulence factors contributing to fungal pathogenicity indicating that the management of infection control unit at Assiut University hospitals must be aware of not only bacterial but also fungal contamination.

## Introduction

Members belonging to the *Aspergillus*, *Fusarium* and Mucorales genera are regarded as the main cause of fungal infections at hospitals (Alberti *et al.,*^2001^; Faure *et al.,*^2000^; Perdelli *et al.,*^2006^). Indeed fungi contaminating hospital rooms may grow on organic matter including various building materials and develop microcolonies. Prior to possible dissemination spores emanating from these colonies could be inhaled by immuno-suppressed patients resulting in local infections (Singh & Paterson, 2005). The ability of fungi to cause human diseases (mycoses) as pathogens appears to be accidental and such diseases are primarily related to the immunological status of the host and environmental exposure, rather than to the infecting organism (Rippon, 1988; Kwon-Chung & Bennett, 1992; Ellis, 1994). A relationship between fungal contamination in hospital environments and the incidence of invasive aspergillosis has been demonstrated (Alberti *et al.,*^2001^) and more than 500 cases of post-operative aspergillosis in immuno-competent individuals have been reported (Pasqualotto and Denning*,*^2006^). *Aspergillus fumigatus* and *A. flavus* are the leading species of the genus *Aspergillus* causing invasive aspergillosis (Pasqualotto, 2008). Outbreaks of nosocomial aspergillosis are attributed to airborne sources and even small concentrations of spores have been associated with outbreaks (Vonberg and Gastmeier*,*^2006^).

Fungi are known to elaborate extracellular enzymes based on the substrate they utilize for growth. Extracellularly produced enzymes have been described in certain fungi such as *Candida* (Borg & Ruchel, 1988) and *Aspergillus* species (Hanzi *et al.,*^1993^). Production and secretion of hydrolytic enzymes, such as proteases, lipases and phospholipases are very important virulence factors. These enzymes play a role in nutrition, tissue damage, fungal dissemination within the human body, iron acquisition and overcoming the host immune system which strongly affects fungal pathogenicity (Ibrahim *et al*., 1995). Secretion of enzymes into extracellular environments might be an important adaptive mechanism during the life cycle of fungi (Monod et *al*., 2002). Earlier studies on fungal enzymatic activities aimed at establishing the role of enzymes in fungal pathogenicity, as well as their capacity to induce inflammatory reactions in the host (Rippon, 1982). It is logical to suppose that these enzymes could act by enabling tissue invasion easier, but they could also participate in causing infection by impairing some mechanisms of the immune system and/or assist in obtaining of nutrients, thus causing injury to the host (Birch *et al.,*^2004^; Da Silva *et al.,*^2005^). Hussein and Brasel *et al.,* (2001) examined the ability of 80 fungal isolates from keratitis patients to produce extracellular enzymes in growth medium and found that most of these isolates could produce protease, lipase, urease and catalase enzymes but at varying levels.

Mycotoxins are an extremely adverse group of low molecular weight fungal secondary metabolites which when ingested, inhaled or absorbed through the skin; can cause lowered performance, sickness or even death in man and animals including birds (Pitt, 1996; and Van Egmond & Speijers, 1999). They may affect the reproductive, immune, gastrointestinal systems, specific target organs, in addition they may exhibit hormonal activity, developmental defects including those relating to birth (tetragenic and neurotoxic) (Richard*,*^1991^*;* Sharma*,*^1993^*;* Kuiper-Goodman*,*^2004^). Although fungal spores may have the highest concentrations of mycotoxins, the vegetative part of the fungus, the mycelium or the substrate upon which the fungus grows can also contain these toxins. Viability of spores is not essential to toxicity. In other words, a dead spore can still be a source of toxin (Keller, *et al.,*^2005^). Infants and children are considered more susceptible to different toxins than adults, because of their lower body weight, higher metabolic rate, incomplete development of some organs and tissues such as those in the central nervous system (WHO*,*^1986^*;* NAS*,*^1993^). Nonetheless, human illnesses caused by mycotoxins may be a public health problem than one realizes because some of these cases (chronic) may go un-noticed for an extended period of time unless large amounts of mycotoxins are consumed resulting in acute symptomology (Hesseltine*,*^1985^). Like the case may be for other hospital infection control units elsewhere, Assiut University hospitals only focus on bacterial infection. This study was directed to examine the potential of the most common fungal species isolated from the atmospheric air and dust samples collected from air conditioners of ICUs and ORs to produce extracellular hydrolytic enzymes and mycotoxic compounds which are important virulence factors involved in fungal pathogenicity.

## Materials and methods

### Screening of fungal isolates for extracellular enzyme production

A total of 110 filamentous fungi isolated from air as well as filters of air conditioning systems in intensive care units and operation rooms (noted in our laboratory) were screened for their ability to produce extracellular enzymes in solid media. The following fungal species were tested: i.e. *Aspergillus flavus* (20 isolates), *A. fumigatus* (16), *A. niger* (19), *Cladosporium cladosporioides* ( 5 ), *Fusarium solani* (17), *F. oxysporum* (6), *Myrothecium roridum* (1) and *Stachybotrys elegans* (26).

Protease activity was determined using a Casein hydrolysis medium in which skim milk gives an opaque final appearance and hydrolysis of the casein resulted in a clear zone around the fungal colony. (Paterson & Bridge, 1994). Lipase activity was measured using the method of Ullman & Blasins (1974) with some modification this time using Tween 80 instead of Tween 20. The lipolytic producing ability was observed as a visible precipitate due to the formation of crystals of calcium salt of the oleic acid liberated by the enzyme. Urease activity was determined using urease medium described by Paterson & Bridge (1994). Isolates capable of producing urease turned the yellow color of the acidic medium to purple-red or deep pink color., meanwhile hemolytic activity of fungal isolates was measured using human blood agar medium (Ronald, 2000).

### Screening of fungal isolates for mycotoxin production

#### Cultivation of fungal isolates and extraction of their mycotoxins

One-hundred and ten isolates were cultivated in a 15 cm Petri dish containing solid Czapek’s glucose agar under aseptic conditions and incubated at 25 ±2°C for 10 days. The plates containing *Fusarium, Stachybotrys* and *Myrothecium* isolates were transferred into a refrigerator for another 10 days. At the end of incubation periods, an agar plug technique was employed for the extraction of mycotoxins in culture materials, whereby, all the agar medium with fungal mycelia were cut into small pieces, transferred into a 250 ml Erlenmeyer flask containing 50 ml 96% methanol. The content was shaken on a rotary shaker (200 r.p.m., 24 h) and filtered through filter paper (Aboul-Nasr; Obied-Allah, 2013).

The extracted material was then washed using 25 ml of the same extractin solvent. The methanol extracts were combined, dried over anhydrous sodium sulphate, and concentrated under a vacuum. The residue was transferred to a dram vial and further evaporated to near dryness.

### Thin layer chromatographic analysis

For the screening of mycotoxins in extracts, a thin-layer chromatographic technique adopted by El- kady and Moubasher (1982) was employed.

## Results and discussion

The ability of clinical fungal strains from various ICUs and ORs at Assiut University hospital units were tested for their ability to produce extracellular enzymes in solid media and data presented in Table 1. Data indicated that more than 66% of tested isolates (73 out of 110) of five fungal genera; 3 *Aspergillus spp*. (55 isolates), one *Cladosporium sp*. (5 isolates), 2 *Fusarium spp.* (23 isolates)*,* one *Stachybotrys sp.* (26 isolates) and one isolate of *Myrothecium sp.* had the ability to produce protease. Nearly similar percent (70.9%) of the tested fungal isolates (78 out of 110) were recorded as lipase producers in this study. Salyers and Witt (1994) reported that microbial cells secrete hydrolytic enzymes that destroy the constituents of host cell membranes leading to membrane dysfunction, physical disruption as well as aid in the invasion of host tissues. Proteolytic degradation of lung tissues has been suggested as one of the key events involved in the physiopathology of *A. fumigatus* (Kothary *et al.,*^1984^). Also, several species of *Aspergillus* such as *A. fumigatus, A. flavus, A. oryzae* and *A. sojae* are known to secrete protease as reported by Monod *et al*., (1993). Stehr *et al*., (2003) found that extracellular lipases play a role during microbial infections and suggested their role is to digest lipids for nutrient acquisition by pathogenic microbe and that these enzymes help the microbe (bacteria or fungi) to grow in environments where lipids are the sole carbon source.

**Table 1:** **Extracellular enzymes produced by the tested fungal isolates collected from ICUs and ORs at Assiut University hospitals**

Fungal isolates	No. of tested isolates	Protease		Lipase		Urease		Hemolysis	
		+ Ve	- Ve	+ Ve	-Ve	+Ve	-Ve	+ Ve	- Ve
***Aspergillus flavus***	**20**	**16**	**4**	**19**	**1**	**20**	**0**	**19**	**1**
***Aspergillus fumigatus***	**16**	**10**	**6**	**15**	**1**	**15**	**1**	**12**	**4**
***Aspergillus niger***	**19**	**15**	**4**	**16**	**3**	**8**	**11**	**13**	**6**
***Cladosporium cladosporioides***	**5**	**1**	**4**	**4**	**1**	**0**	**5**	**2**	**3**
***Fusarium solani***	**17**	**11**	**6**	**9**	**8**	**10**	**7**	**7**	**10**
***Fusarium oxysporum***	**6**	**3**	**3**	**5**	**1**	**5**	**1**	**4**	**2**
***Myrothecium roridum***	**1**	**1**	**0**	**1**	**0**	**1**	**0**	**1**	**0**
***Stachybotrys elegans***	**26**	**22**	**4**	**25**	**1**	**19**	**7**	**19**	**7**
**Total**	**110**	**79**	**31**	**94**	**16**	**78**	**32**	**77**	**33**

A majority of the fungal isolates under study (92 out of 110: 83.6%) were able to produce urease. Urease catalyses the hydrolysis of urea to ammonia and carbamate, in which the latter by-product is further hydrolyzed to ammonia and carbonic acid resulting in an increase in pH (Zimmer, 2000). Urease activity has been found in several bacteria and fungi and has been shown to be an important pathogenic factor (Eaton *et al*., 1991; Cox *et al*., 2000). It has also been postulated that much of the tissue damage induced by *Helicobacter pylori* is as a result of ammonium hydroxide produced through the actions of urease. Studies have shown that the actions of urease may alter the function of white blood cells (Mai *et al.,*^1992^*;* Mobley, 1996). In this study, about 70.9% of the tested fungal isolates (78 out of 110) exhibited a lysis activity (heamolysis) on human blood. Vesper *et al.* (1999; 2001) isolated stachylysin a hemolytic agent from *Stachybotrys chartarum.* ( Donohue *et al.,*^2004^) isolated chrysolysin another hemolytic agent from *P. chrysogenum.*

The toxigenic potentials of these fungal isolates previously tested herein for their enzymatic activity were also evaluated and the data presented in Table 2 revealed that 79 out of the 110 tested fungal isolates (71.82%) were recorded as mycotoxin producers. Several mycotoxins including aflatoxins, gliotoxin, fumigillin, cladosporin, T-2 toxin, zearalenone, roridins, verrucarins, trichoveroides and satratoxins were produced by different fungal isolates in this study. Aflatoxins B_1_ & B_2_ were recorded in extracts obtained from cultures of 11 *A. flavus* with one positive isolates being able to produce additionally aflatoxins G_1_ & G_2_. Aflatoxins are produced by many strains of *A. flavus*. They are toxic, having carcinogenic, mutagenic and teratogenic effects in laboratory animals (Abdel-Wahhab *et al.,*^1998^,^2006^). Aflatoxin B_1_ is the most potent carcinogenic substance naturally produced mainly by *A. flavus* (Squire, and *A. parasiticus*^1981^) and is classified by the International Agency of Research on Cancer as a group 1 human carcinogen (IARC, 1982). Gliotoxin and fumigillin were produced by all the 16 tested isolates of *A. fumigatus*. Also, 7 out of 19 tested isolates of *A. niger* had the ability to produce gliotoxin. *Aspergillus* members especially *A. fumigatus* and *A. niger* are the most common causal agents of aspergillosis. Invasive aspergillosis is very common among immuno-compromised patients, with reported incidence rates of 17-26% among lung transplants patients, 5-15% allogenic bone marrow transplants patients, 5-24% of those with acute leukaemia and 2-13% heart transplants patients (Curtis *et al.,*^2004^). Kupfahl *et al.,* (2008) investigated the presence of gliotoxin-producing *Aspergillus* strains among clinical isolates collected from different parts of Germany and Austria. In that light, we collected and examined 158 different *Aspergillus* isolates consisting of 100 *A. fumigatus*, 27 *A. terrus*, 15 *A. flavus* and 16 *A. niger* strains from different medical centers and other environmental samples. According to that study, gliotoxin was recovered from 98, 56, 37 and 13% of *A. fumigatus, A. niger, A. terreus* and *A. flavus,* respectively. Nielsen (2003) found that *A. fumigatus* and *A. niger* are frequently isolated as indoor moulds, with the former isolate having the ability to produce fumitoxins, fumitremergens, gliotoxin and other mycotoxins, while the later produced only ochratoxin A. Cladosporin was produced by five tested isolates of *C. cladospoioides* in this study. This species is one of the causative agents of skin lesions, keratitis, nail fungus and pulmonary infections. Also, acute symptoms of exposure to this fungus are edema and bronchiospasr which may lead to pulmonary amphysema. Zearalenone was produced by all six tested isolates of *F. oxysporum* and three out of 17 isolates of *F. solani.* This toxin has an estrogenic activity and this estrogenic property enables exposure to its products a concern for human health (Niyo *et al*., 1988, Richard, 1991). In humans, the major effect of zearalenone bears on the reproductive system affecting reproductive organs' structure and function that may lead to hyperestrogenism (Kuiper-Goodman *et al.,*^1993^). T-2 toxin productivity by 11 of the 17 tested *F. solani* isolate was recorded in this study. This toxin is a representative of a large group of non-macrocyclic trichothecenes whose major effect and that of other trichothecenes is their ability to inhibit protein synthesis which is followed by a secondary disruption of DNA and RNA synthesis (Niyo *et al*., 1988; Richard, 1991). Verrucarin A & J as well as roridin A & E are mycotoxins produced by as *M. roridium* found in this study*.* Also, verrucarin J was produced by 11 out of 26 tested isolates of *S. elegans* (Eppley, with 5 of this species found to produce satratoxins H & E. Meanwhile 19 of the same species were able to synthesize trichoveroides.Verrucarins, roridins, trichoveroides and satratoxins. Different types of macrocyclic trichothecenes produced mainly by *Stachybotrys*^1977^; Eppley and Bailey, 1973; Eppley *et al.,*^1980^). The involvement of macrocyclic trichothecenes in stachybotryotoxicosis were suggested by isolation of satratoxins from straws fed to sheep and cattle with stachybotryotoxicosis (Harrach and Bata, 1983; Harrach *et al.,*^1983^). Bata *et al.* (1985) found that all 17 strains of *Stachybotrys atra* ( isolated in Middle Europe and found to produce each of the five macrocyclic trichothecenes that included satratoxins H & G, verrucarin A, E & J, trichoveroides and roridin A & E. Islam *et al.,*^2006^) suggested that neurotoxicity and inflammation in the nose and brain are potential adverse health effects of exposure to satratoxins.

**Table 2:** **Mycotoxigenicity of fungal isolates collected from ICUs and ORs at Assiut University hospital**

Toxins produced	No. of strains positive	No. of strains tested	Fungal isolates
**Aflatoxins B1, B2**	**10**	**20**	***Aspergillus flavus***
**Aflatoxins B1, B2, G1, G2**	**1**		
**Gliotoxin, fumigillin**	**16**	**16**	***Aspergillus fumigatus***
**Gliotoxin**	**7**	**19**	***Aspergillus niger***
**Cladosporin**	**5**	**5**	***Cladosporium cladosporioides***
**T-2 toxin**	**9**	**17**	***Fusarium solani***
**T-2 toxin, Zearalenone**	**2**		
**Zearalenone,**	**1**		
**Zearalenone**	**6**	**6**	***Fusarium oxysporum***
**Roridin A&E, Verrucarin A&J**	**1**	**1**	***Myrothecium roridum***
**Trichoveroides**	**10**	**26**	***Stachybotrys elegans***
**Trichoveroides&verrucarin J**	**6**		
**Satratoxin H&E, Trichoveroids, Verrucrin J**	**5**		

## Conclusion

In conclusion, hydrolytic enzymes and mycotoxic compounds which are considered the most important virulence factors influencing the pathogenicity of opportunistic fungal infections were detected in most of the cultures of fungal isolates tested herein. Thus the personnel managing the infection control unit of Assiut University hospitals must be aware of not only bacterial contamination, but there is a potential for the distribution of fungal infection as well.
